# Hypereosinophilic Syndrome Leading to Severe Right-Sided Heart Failure in a Patient with Ebstein's Anomaly

**DOI:** 10.1155/2013/659832

**Published:** 2013-05-23

**Authors:** Joas John Kyabonaki, Bjarne Linde Nørgaard, Søren Høyer, Niels Holmark Andersen

**Affiliations:** ^1^Department of Cardiology, Aarhus University Hospital, 8200 Aarhus, Denmark; ^2^Department of Pathology, Aarhus University Hospital, 8000 Aarhus, Denmark

## Abstract

A 36-year-old male with mild Ebstein's anomaly developed severe right-sided heart failure, following a 5-year-long course of hypereosinophilic syndrome. No regular followups had been done, during the years of antineoplastic therapy. A year after being cured from the hypereosinophilic syndrome, the patient developed right-sided heart failure symptoms and was found to have excessive fibrosis of the right ventricular endocardium and free tricuspid regurgitation. The findings were compatible with substantial scarring of the endocardium caused by the hypereosinophilic syndrome. Over a few years, the patient deteriorated significantly and was finally offered a heart transplant. Examination of the explanted heart revealed severe fibrosis of the right ventricle and almost complete sparing of the left.

## 1. Introduction

The hypereosinophilic syndromes (HES) are a group of disorders, with sustained overproduction of eosinophils, in which eosinophilic infiltration can cause damage to multiple organs [1]. The overall prevalence of myocardial involvement is more than 50% and can be related to a poor prognosis [1, 2]. Typical cardiac manifestations are endocardial fibrosis and mural thrombosis, predominantly located in the apices of both ventricles [2–4]. The long-term prognosis depends on the degree of cardiac involvement and the likelihood of hematological malignancies later on [1]. However, cardiac disease from HES only rarely leads to end-stage heart failure or heart transplantation [5].

## 2. Case Report

A 36-old-male patient was referred to our department, due to severe tricuspid valve regurgitation and right-sided heart failure. At age of 29, he was diagnosed with HES and treated with hydroxyurea and prednisolone for 5 years. The patient had never had cardiac symptoms before initiation of the antineoplastic therapy, and a pretreatment echocardiogram had only shown mild Ebstein's anomaly, with mild tricuspid valve regurgitation.

**Figure 1:**
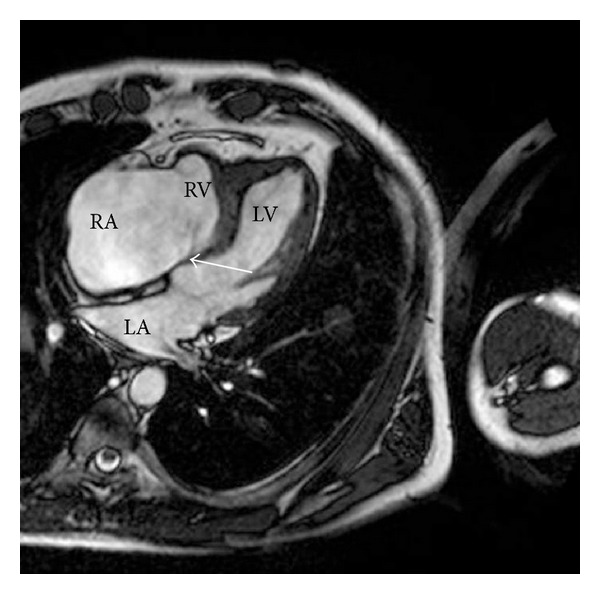
Cardiac MRI shows dilatation of the right atrium and severe tricuspid regurgitation. Small right ventricular cavity and slightly displaced offset of the tricuspid valve are seen, characteristics for Ebstein's anomaly (arrow). RA: right atrium; RV: right ventricle; LA: left atrium; LV: left ventricle.

**Figure 2:**
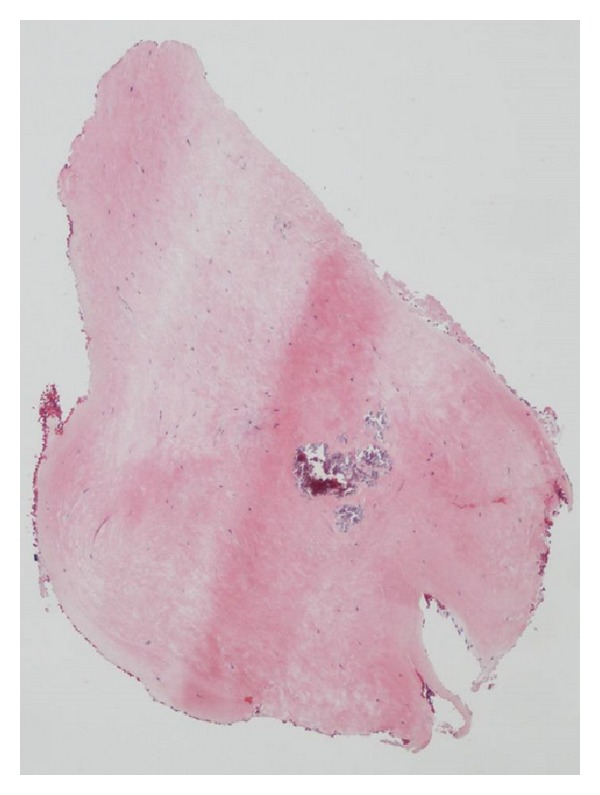
Microphotography of endocardial biopsies. The biopsies only demonstrated fibrosis and no eosinophilia.

Maybe for these reasons, the patient was not offered any cardiological check-ups during the course of HES. Approximately one year after being declared free of HES, including the *FIP1 L1-PDGFRA* gene rearrangement, the patient developed right-sided heart failure symptoms. A new echocardiogram revealed severe tricuspid regurgitation with dilatation of the right atrium. The right ventricle was found restrictive with poor systolic function; however, the acoustic windows were poor. A subsequent cardiac magnetic resonance scan revealed a small right ventricular cavity, poor systolic right ventricular function, and agglutination of the tricuspid valve leaflets, causing severe regurgitation and paradoxical movement of the interventricular septum (Figure 1). The patient underwent a heart catheterization which indicated ventricularization of the right atrium and a decreased cardiac index of 1.7 L/min/m^2^. A routine coronary angiogram showed insignificant coronary artery disease (the patient was a previous smoker). Endomyocardial biopsies from of the right ventricular endocardium were obtained with great difficulty, all with severe fibrosis but without eosinophilia (Figure 2). No pulmonary affection was ever found.

The patient was treated with heart failure medication and followed closely but deteriorated to severe heart failure over a 5-year period, leading up to a new complete cardiac evaluation.

**Figure 3:**
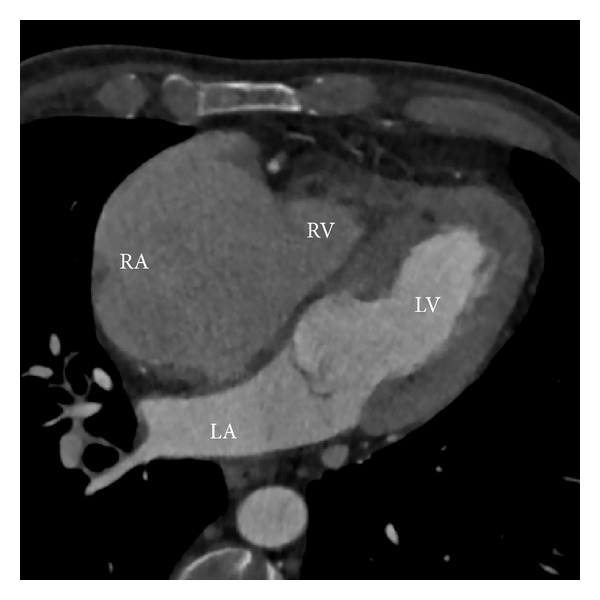
Cardiac CT five years after the MRI scan. The right atrial cavity is now markedly enlarged, and the right ventricular cavity is almost obliterated. The left-sided cavities appear normal. RA: right atrium; RV: right ventricle; LA: left atrium; LV: left ventricle.

A new echocardiogram revealed restrictive filling of the right ventricle, almost free tricuspid regurgitation, and massive right atrial enlargement. Due to poor acoustic windows, a CT scan was done (a repeated MRI was not possible due to claustrophobia). The CT scan showed almost obliteration of the right ventricular cavity and massive dilatation of the right atrium (Figure 3). The patient's oxygen consumption was now very poor (12.9 mL/kg/min), and a cardiac catheterization showed a very low cardiac index (1.5 L/min/m^2^). The patient was listed for a heart transplant and successfully transplanted 3 months later. 

**Figure 4:**
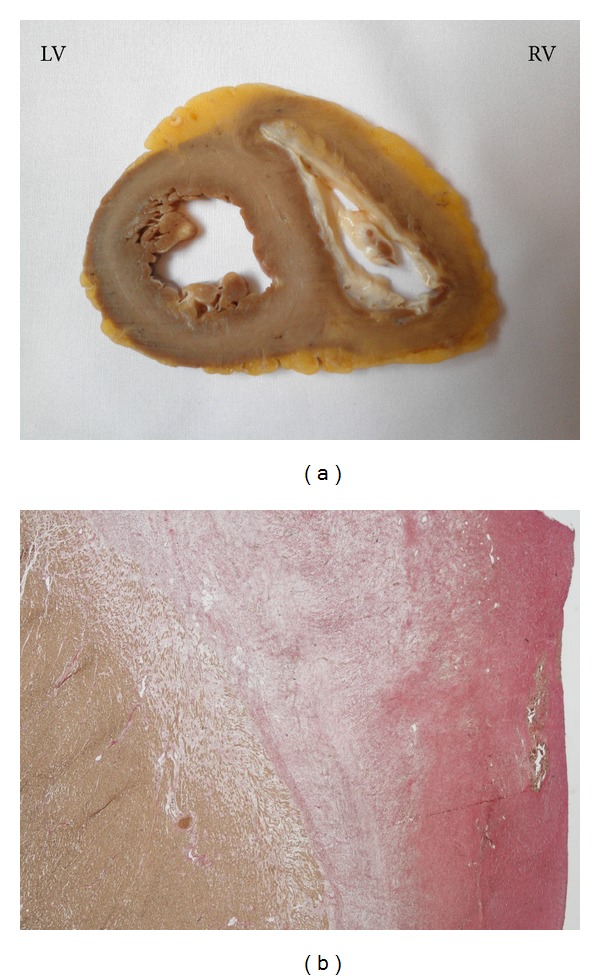
(a) Midventricular cross-section of the ventricles, showing a pronounced endocardial thickening of the right ventricle and a tiny right ventricular cavity. On the left side, only mild endocardial thickening and subendocardial fibrosis are evident. RV: right ventricle; LV: left ventricle. (b) Microphotography of the endocardium of the right ventricular septum, with minor calcification and diffuse subendocardial fibrosis.

Detailed investigations of the explanted heart revealed excessive fibrosis of the right ventricle with circumferential thickening of the right ventricular endocardium, almost obliterating the right ventricular cavity (Figure 4). The left ventricle only showed mild endocardial thickening and subendocardial fibrosis (Figure 4). Microscopic examination showed massive fibrosis without eosinophilia (Figure 4). 

Two years after the heart transplant, the patient is doing well, without any signs of eosinophilia.

## 3. Discussion

The early stage of cardiac involvement in HES begins with eosinophilic infiltration of the endomyocardium, followed by an intermediate thrombotic stage [4], and a late fibrotic stage resulting in endomyocardial fibrosis [2, 3]. In the fibrotic stage, progressive scarring causes restrictive cardiomyopathy and/or AV-valve regurgitation, due to entrapment of the chordae tendineae [2, 6].

In the present case with Ebstein's anomaly, there was a predilection for myocardial involvement of the right ventricle and the tricuspid valve, causing severe deterioration of the tricuspid valve function and progressive heart failure, while the left ventricle was almost spared.

It is likely that the mild Ebstein's anomaly was decisive for the progressive nature of this case; however, this issue remains unresolved.

Since the patient was without cardiac symptoms during the disease stage with hypereosinophilia, myocardial involvement was not suspected. Therefore, no regular examinations were performed. It is far from certain that this would have changed the final course. However, it would have been useful to be aware of cardiovascular complications to this rare hematological disease, when they appeared.

For this purpose, cardiovascular magnetic resonance (CMR) imaging may have been a useful tool, since myocardial involvement often presents with positive subendocardial late gadolinium enhancement [3]. However, there is no general recommendation for CMR during the course of HES [7]. Instead, echocardiography is mentioned for risk assessment [7], but even though echocardiography is widely available and useful for functional and anatomical evaluation, it cannot provide appropriate soft tissue characterization to be absolutely sure whether there is cardiac involvement or not [3]. In the present case, poor echocardiographic windows made this even more difficult. To be absolutely certain of the diagnosis, endomyocardial biopsy is the gold standard for establishing whether the myocardium is affected or not [6].

The present case represents the final fibrotic stage of cardiac involvement, involving irreversible heart failure. It also clearly shows that even in the absence of symptoms, cardiac involvement should be highly suspected in a patient with HES.

Whether a more aggressive treatment regimen would have changed the final course in this case will never be determined.
